# LED-Induced fluorescence and image analysis to detect stink bug damage in cotton bolls

**DOI:** 10.1186/1754-1611-7-5

**Published:** 2013-02-20

**Authors:** Adnan Mustafic, Erin E Roberts, Michael D Toews, Mark A Haidekker

**Affiliations:** 1 College of Engineering, University of Georgia, Athens, 30602-4435, GA, USA; 2 Department of Entomology, University of Georgia, Tifton, 31793-0748, GA, USA

## Abstract

**Background:**

Stink bugs represent a major agricultural pest complex attacking more than 200 wild and cultivated plants, including cotton in the southeastern US. Stink bug feeding on developing cotton bolls will cause boll abortion or lint staining and thus reduced yield and lint value. Current methods for stink bug detection involve manual harvesting and cracking open of a sizable number of immature cotton bolls for visual inspection. This process is cumbersome, time consuming, and requires a moderate level of experience to obtain accurate estimates. To improve detection of stink bug feeding, we present here a method based on fluorescent imaging and subsequent image analyses to determine the likelihood of stink bug damage in cotton bolls.

**Results:**

Damage to different structures of cotton bolls including lint and carpal wall can be observed under blue LED-induced fluorescence. Generally speaking, damaged regions fluoresce green, whereas non-damaged regions with chlorophyll fluoresce red. However, similar fluorescence emission is also observable on cotton bolls that have not been fed upon by stink bugs. Criteria based on fluorescent intensity and the size of the fluorescent spot allow to differentiate between true positives (fluorescent regions associated with stink bug feeding) and false positives (fluorescent regions due to other causes). We found a detection rates with two combined criteria of 87% for true-positive marks and of 8% for false-positive marks.

**Conclusions:**

The imaging technique presented herein gives rise to a possible detection apparatus where a cotton boll is imaged in the field and images processed by software. The unique fluorescent signature left by stink bugs can be used to determine with high probability if a cotton boll has been punctured by a stink bug. We believe this technique, when integrated in a suitable device, could be used for more accurate detection in the field and allow for more optimized application of pest control.

## Background

The southern green stink bug (*Nezara viridula* L.) (Hemiptera: Pentatomidae) is one example of a stink bug that feeds on and thereby damages plants. This species is thought to originate from Ethiopia, but is now found in many tropical and subtropical regions of the world. This species is commonly found in the southern United States where it is considered a major pest attacking agricultural crops. Stink bugs feed on a variety of wild and cultivated plants and crops including but not limited to legumes, nuts, and various fruits and vegetables [1-4]. One of the extensively planted crops in the southeastern U.S. susceptible to stink bug infestation is cotton (*Gossypium hirsutum* L.) [1]. Most stink bugs feed on developing cotton bolls, which occur after anthesis. Both nymphs and adults are capable of feeding and damaging crops, and mature nymphs tend to cause more damage than younger instars [5].

The feeding mechanism of stink bugs includes the extension of a piercing mouthpart (proboscis), with which it penetrates the outer wall to reach the developing seeds. Pathogenic bacteria may be introduced during feeding, thereby adding an additional destructive agent resulting in further degradation of bolls, especially in less mature bolls [6,7]. Cotton bolls damaged by stink bugs result in reduced fiber length, quality, and uniformity [8-10]. Another effect of stink bug feeding on cotton bolls is the lower rate of seed germination [11]. These types of damage greatly diminish the value of cotton lint and can cause substantial impediments to a stable cotton supply. The most common approach to reduce stink bug damage is to wait until damage exceeds an economic threshold and then apply insecticides to mitigate the population.

While highly specific insecticides and genetic engineering of the host crop have become preponderant methods in agricultural pest control, these methods have not been effective against stink bugs. Therefore, stink bugs are generally managed using broad spectrum insecticides, such as pyrethroids and organophosphates, that kill the intended pests but also many non-target beneficial insects. Proper timing and application of insecticides is critical to reduce the risk of secondary pest outbreaks [12,13]. To prevent financial losses, insecticides need to be applied only when the estimated damage exceeds an economic threshold. Moreover, the choice of insecticide chemistry can have a profound effect on efficacy. Along with choosing appropriate types of insecticides for application, timing is important, because organophosphates and pyrethroids have limited residual activity in the field. In other words, insecticides need to only be applied at the correct time and place or the grower risks erosion of financial gains.

It is of essential importance to continue research in this field because of the economic importance of cotton. The United States are the world’s largest exporter of cotton with 11.6 billion bales per year in 2012, which is nearly three times more than the next closest country, Australia, with 4.25 billion bales per year [14]. The southeastern United States, which is the production region with the most stink bug damage, produces approximately 25% of the US upland cotton crop [15].

To develop an appropriate plan for pest control in the field, two requisite and codependent challenges regarding sampling and damage detection exist. The currently recommended practice of sampling cotton bolls is time consuming, burdensome, and destructive to the test population of cotton bolls. Recent studies describe various issues with monitoring stink bug populations which are predisposed to aggregate in fields and can be difficult to detect with the naked eye [16,17]. Even with optimized sampling plans, cotton boll examination for potential damage is an invasive procedure which renders the bolls useless because it requires them to be cracked open. Development of a new method with proven accuracy and efficiency to detect stink bug feeding is necessary.

In this article, we propose an image analysis technique that builds on previous research by Xia *et al.*[18]: the presence of different fluorescence emission was reported, depending on whether the cotton boll was damaged by stink bugs or not. Stink bug feeding results in a unique fluorescent signature on the cotton boll wall that can be imaged and analyzed for discriminating properties. One of the advantages of this technique is that it is non-invasive and nondestructive. Here, we report on an image segmentation and analysis process capable of distinguishing feeding marks from stink bugs versus those caused by other factors.

## Methods

### Stink bug rearing

Southern green stink bugs were reared in the laboratory on a diet of fresh green beans or okra pods following the methods of Harris and Todd [19]. Briefly, adult stink bugs captured in the field were brought into the lab and placed in 37.9 liter glass aquaria lined with paper towels. Adults oviposited on the paper towels. These were transferred to ventilated petri dishes until the eggs hatched. Nymphs were maintained in petri dishes or small plastic wide-mouth jars at 25.0°C and 65% relative humidity. Adults used for infesting bolls were less than two weeks old and of mixed sex.

### Cotton boll development

Cotton plants were started from seed and grown in the greenhouse to produce bolls for the study. Three cotton seeds (FM 9063 B2RF) per 11.35 liter plastic pot were sewn in Metro Mix 300 growing medium. After germination, two of the plants were culled and the remaining plant was fertilized monthly with Osmocote 14-14-14 and Micromax fertilizer (the Scotts Co. LLC, Marsville, OH). Once the plant began flowering, individual white flowers were marked daily using flagging tape and then allowed to develop normally for 10 to 14 days. When the bolls reached 10 to 14 d past anthesis, a single southern green stink bug adult was caged in a mesh bag on each boll and subtending leaf for a period of 48 h.

After treatment with the stink bug, bolls were excised from the plant and shipped with overnight service to the Athens campus for imaging. At the time the bolls were cut from the plant they had an external boll diameter of 2.3 to 2.5 cm. Control bolls were treated exactly as described, except that no stink bugs were introduced into the mesh bags. A total of 136 bolls were harvested that were exposed to a stink bug, and 109 control cotton bolls were harvested.

### Mechanically punctured cotton bolls

Sixteen additional cotton bolls were punctured with a sterile syringe needle (31 Ga, 8mm long, Beckton-Dickinson, product no. 328418) as a control. These bolls were treated exactly as described in the previous section with two differences: (a) no stink bugs were introduced into the mesh bag. and (b) before harvesting, the bolls were gently cleaned using rubbing alcohol on a cotton swab to remove any microorganisms that could contaminate the boll. Puncturing was done manually, and care was taken that the needle penetrated into the lint tissue. The punctured bolls were harvested immediately. Bolls with needle punctures were processed in the same manner as the other bolls. The purpose of these controls were to examine the difference between purely mechanical puncture damage and the specific puncture damage caused by stink bugs.

### Imaging apparatus

**Figure 1:**
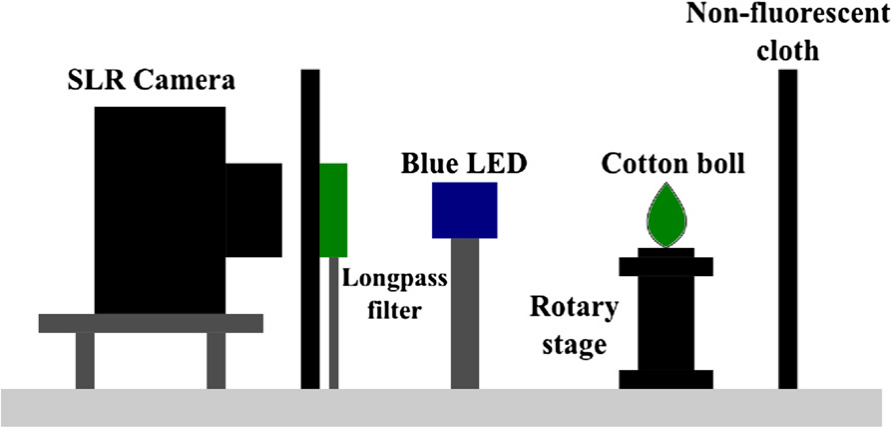
**Side view of the imaging apparatus.** A SLR camera is mounted on posts in front of the sample, with a dichroic emission longpass filter placed between sample and lens. Two blue LED sources (at 45°angle from the lens-sample path) provide homogeneous excitation light. The cotton boll sample is placed on a rotary stage to allow images to be taken from all sides. A non-fluorescent black cardboard backdrop blocks scattered light and background fluorescence.

The imaging apparatus, shown in Figure 1, consisted of a consumer-grade single lens reflex (SLR) digital camera (Konica Minolta, Dynax Maxxum 7D, Tokyo, Japan) with a Sigma 50 mm f/2.8 fixed focus lens. An emission longpass filter (490nm, Chroma Technology Corp., Brattleboro, VT) was positioned on a post in front of the camera. Two LED sources were available. First, a high-intensity 370nm LED area illuminator (Edmund Optics NT59-369, center wavelength 370nm, FWHM 35nm, driver current 500mA) provided near-UV light similar to the broad-band UV source used in [18]. Second, a dual deep blue LED excitation source (Philips/Luxeon LXHL-LR5C with custom LED driver) provided continuous and homogeneous excitation light at 440nm with a full-width half-maximum bandwidth of 20nm. Additional excitation bandpass filters D450/40 (Chroma) were placed on the blue LED to reduce the spectral overlap between excitation and emission light and limit the emission spectral range to 420nm – 460nm. A rotary stage held cotton bolls during imaging. A non-fluorescent black cardboard behind the sample stage reduced background fluorescence and scattered light.

### Image acquisition and processing

All images were acquired with lens aperture set at f/11 and an exposure time of 15 seconds. All automatic camera functions were disabled, the white balance was fixed to 5500 K, camera sensitivity kept at ISO 400, and the camera set to acquire raw images in MRW (Minolta Raw) format. Each cotton boll was placed on the rotary stage and rotated 90° after each image acquisition, resulting in four images per cotton boll. Raw-format images have a depth of 12 bits per pixel, and all images were converted with UF-Raw ( http://ufraw.sourceforge.net) to 16-bit TIFF images with the 12-bit depth fully retained.

**Figure 2:**
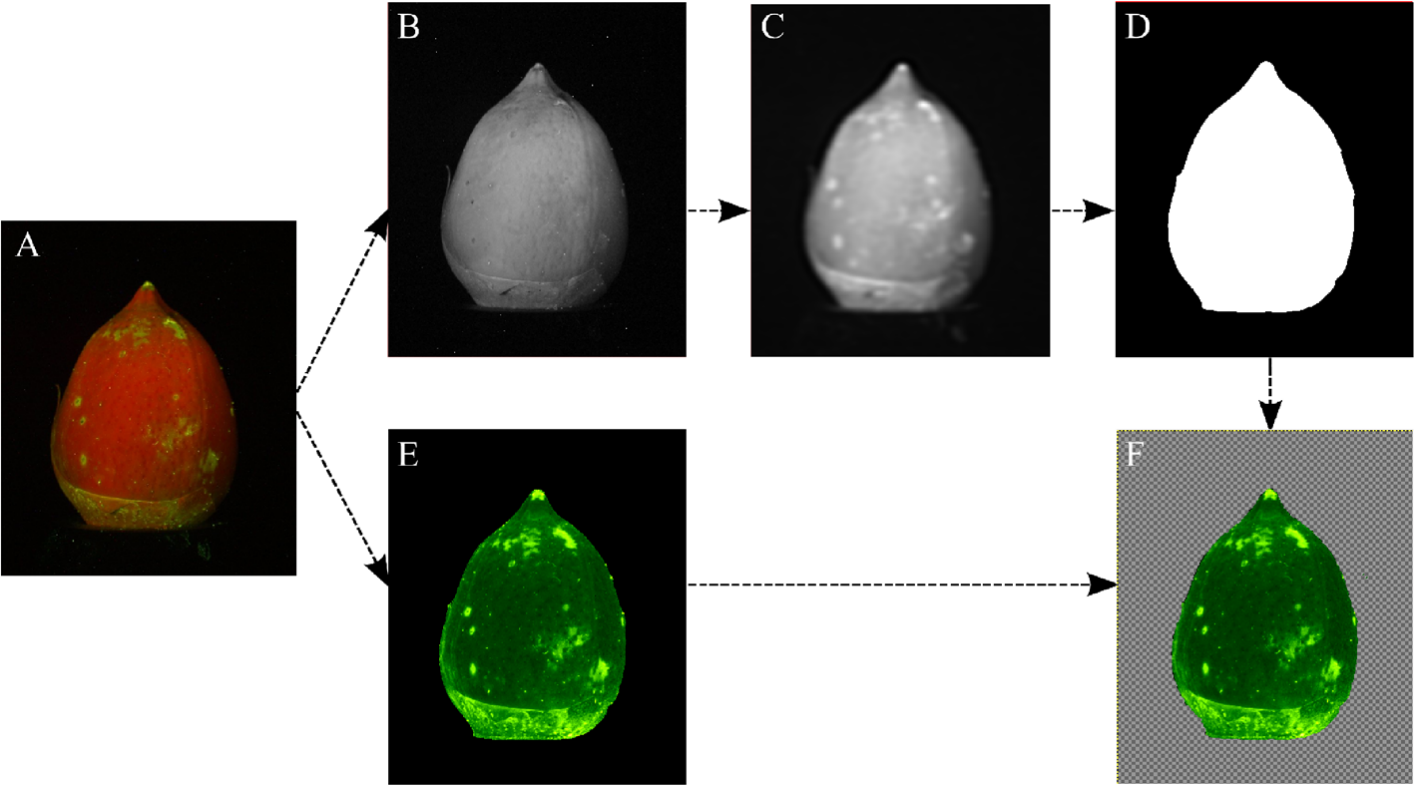
**Processing steps of cotton boll images.** A native camera raw image (**A**) is separated into the monochromatic R, G, and B channels, and the B channel is discarded. To obtain the cotton boll mask, the green channel is filtered and reduced in size (**B**), then further blurred with a Butterworth lowpass filter (**C**). The mask is then obtained by thresholding (**D**). In parallel, a normalized intensity is generated by dividing the green channel by the red channel on a pixel-by-pixel basis (**E**). Multiplication with the mask yields the final image of normalized green intensity from the cotton boll, separated from background (**F**). The background in (F) is indicated by the gray checkerboard pattern and is excluded from any image analysis steps.

All further image processing was performed with Crystal Image [20], a quantitative image analysis software. The images were first subjected to a center-weighted median filter, and their resolution reduced to 1508 by 1004 pixels with a 2 × 2 binning operation. The reduced-scale images showed a resolution of 18 pixels/mm. After the initial steps, two different paths were taken: Mask creation, and computation of a ratiometric image. For the mask, the red channel was blurred with a second-order Butterworth lowpass filter with a cutoff of 10 pixels^−1^. Background separation was performed with a fixed threshold, made possible by the black cloth backdrop. The resulting mask contained the image value 1 for all pixels that correspond to the cotton boll, and 0 for all background pixels. The ratiometric image was computed by extracting the red and green channels from the image and dividing the green channel by the red channel on a pixel-by-pixel basis. Subsequent multiplication of this intermediate image with the mask, also on a pixel-by-pixel basis, yielded an image that contained zero-valued pixels for the background and the green/red intensity ratio for the cotton boll pixels. The image processing steps are illustrated in Figure 2.

Candidates for stink bug puncture marks were identified with a local maximum filter with the *a priori* settings of a peak-to-peak distance of 15 or more pixels and a minimum intensity difference of 10 to the average inside a 15-pixel radius and segmented with a variant of the greedy snake algorithm [20]. This snake algorithm is a special variant of active contour models. A circle is placed near the fluorescent mark that is larger than the actual mark, and whose placement is uncritical. The circle is discretized into 32 vertices, and the vertices are iteratively displaced to minimize the total snake energy. The snake energy is defined as the sum of the vertex distance (stretching energy), the path first derivative (bending energy) and the negative image gradient (external energy). With this energy formulation, the snake tends to contract in a regular (i.e., circular fashion) until it locks onto the image gradient, and the iterative displacement ends near the gradient maximum of the boundary of the fluorescent mark.

**Figure 3:**
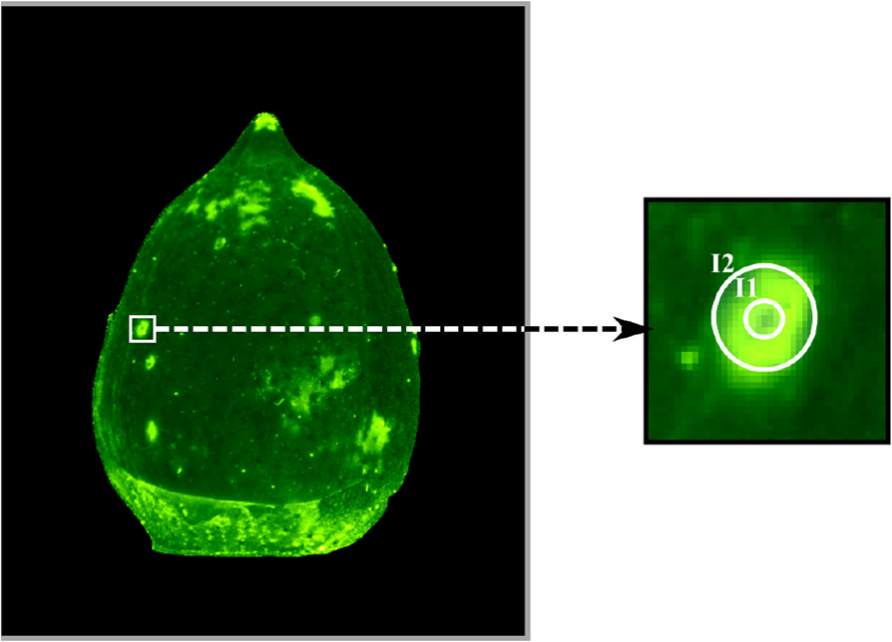
**An example of how fluorescent surface damage marks on cotton bolls were selected and evaluated for analysis.** Strongly fluorescent regions, identified with a local maxima filter, were analyzed with respect to area and intensity. One representative region has been magnified, and the two auxiliary circular regions indicated. Average intensity inside the smaller circle is denoted *I*_1_, and average intensity inside the larger circle is denoted *I*_2_. In instances where stink bugs or syringe needles made the puncture, the ratio of *I*_1_/*I*_2 _often falls below 1. On the other hand, when damage marks were found on non-infested cotton bolls, the ratio of *I*_1_/*I*_2 _was less than 1 in only 24% of cases.

Fluorescent marks were excluded from analysis when their area was below 28 pixels (0.09 mm^2^) or their integrated intensity was below 0.04 mm^−2^. Furthermore, all fluorescent marks with an aspect ratio greater than two or with a convex shape were excluded. The snake was then converted into a circle, and the average intensity *I*_2 _was measured inside this circle. A smaller concentric circular region with a radius of 4 pixels (≈ 0.05 mm^2^) was then selected to compute the central average intensity *I*_1_. A ratio *I*_1_/*I*_2_ was computed for each candidate for stink bug puncture marks (Figure 3).

### Statistical analysis

Statistical analysis was performed in R ( http://www.r-project.org). The ability of the area metric and the intensity metric to distinguish true and false positives was tested individually with the Kruskal-Wallis test and Dunn’s multiple comparison test. The choice of the Kruskal-Wallis test over one-way ANOVA was dictated by the nonparametric distribution of the data. Deviation of a sample’s median value from a hypothetical value was tested with the Wilcoxon signed rank sum test. A significance level of *α *= 0.05 was assumed.

## Results and discussion

The purpose of this study was to provide proof-of-principle that LED-induced fluorescence combined with image analysis can provide an indicator whether a cotton boll has been fed on by stink bugs or not. Here, the most important component was the ability to detect stink bug-related fluorescent marks on the outer carpal wall without the need to manually open the boll.

The starting point for this study is an earlier publication [18], in which we reported on the observation of a characteristic green fluorescence emission near stink bug puncture sites. This fluorescence was prominently visible with the unaided eye under ultraviolet illumination on the lint and the inner carpal wall. We also observed that some fluorescence becomes visible on the outer carpal wall, while at the same time the red chlorophyll emission recedes.

**Figure 4:**
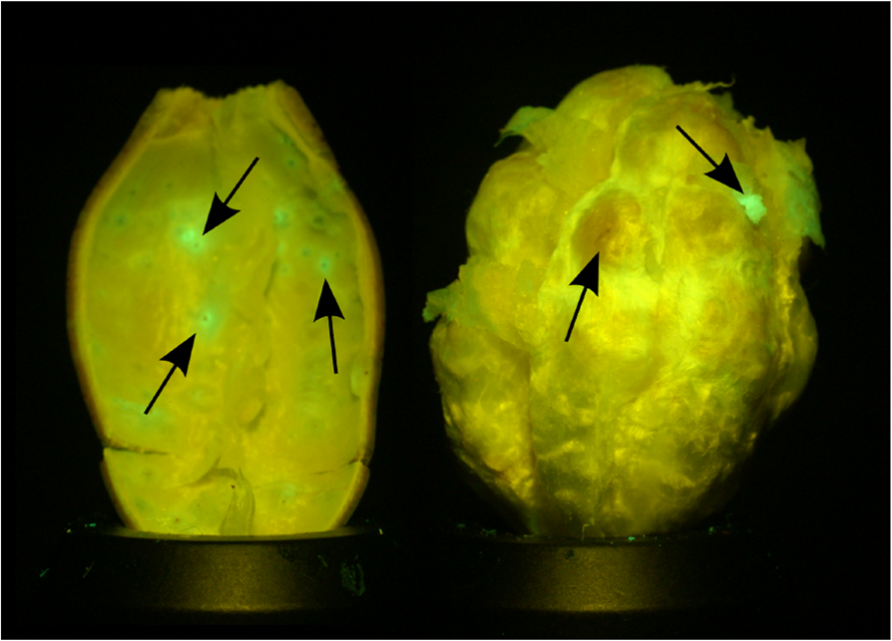
**A fluorescent image of the cotton boll carpal wall (left) and cotton boll lint (right) with arrows denoting the damage caused by stink bugs.** The carpal wall shows fluorescent interior warts, which often have a noticeable dark center where the stink bug pierced the wall. Stink bug damage to the lint is visible in two different ways: lint browning (visible under regular white illumination) and fluorescent damaged regions that correspond with the piercing marks on the carpal wall.

Further examination of this phenomenon revealed that the stink bug marks on the outer carpal wall are more prominently visible under deep blue excitation than under ultraviolet excitation: Contrast of verified puncture marks, defined as the difference between green intensity and red (chlorophyll) intensity normalized by red intensity, was typically increased four-fold under deep blue illumination when compared to UV illumination. For this reason, any further image acquisition was performed with LED excitation at 440nm. The fluorescence emission that accompanies typical damage on the inside of a cotton boll is shown in Figure 4.

Any bolls that were punctured with a sterile needle exhibited a similar fluorescent pattern on the outside carpal wall and wart development on the inner carpal wall, although the outside diameter of a 31 gauge needle (0.26mm) is larger than a stink bug proboscis, estimated at 0.17 mm [21]. Unlike in bolls punctured by stink bugs, lint coloration and the later development of shriveled and dried lint as well as mold was absent. We conclude that the fluorescence is a by-product of the plant healing process, but that stink bug feeding introduces additional damage due to pathogen contamination. For the image analysis process, however, this observation is of no relevance.

**Figure 5:**
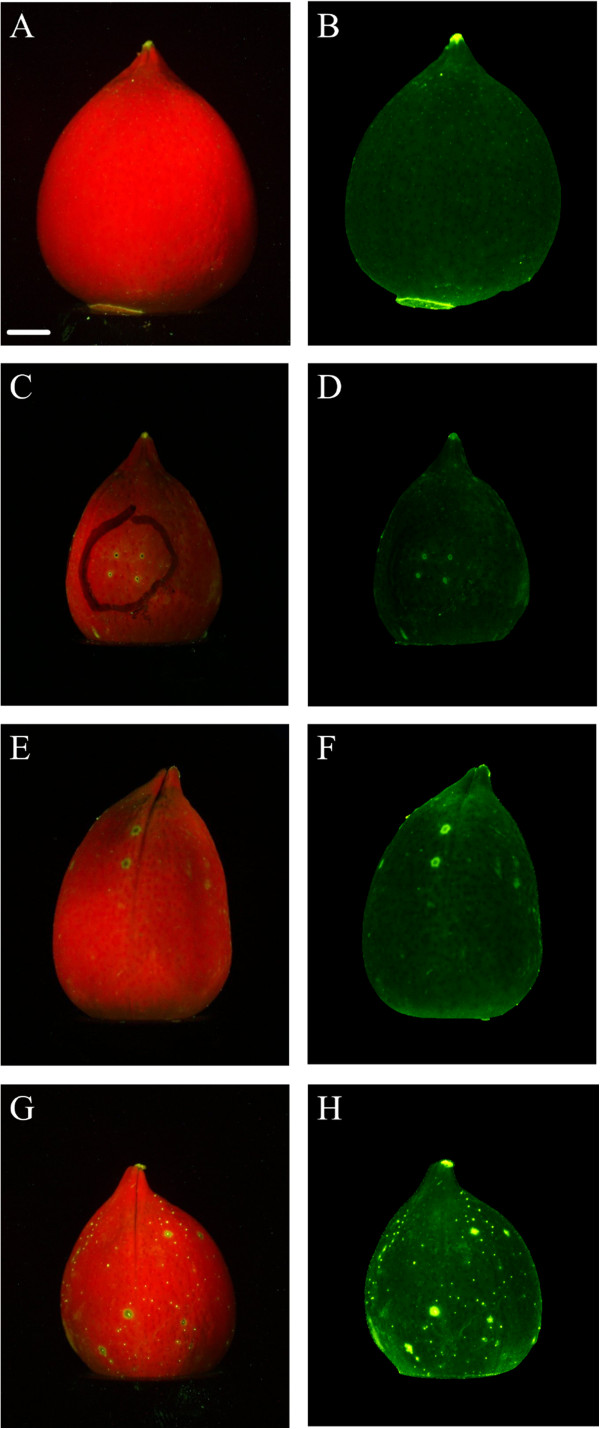
**Raw color images (left column) and corresponding ratiometric images (right column) of cotton bolls with various types of damage and stages of infestation.** (**A**) and (**B**) show a non-infested cotton boll without any exterior damage marks. (**C**) and (**D**) show a cotton boll punctured by a sterilized syringe for negative control studies. The black ring in (C) was drawn with a fiber tip marker. (**E**) and (**F**) show an example of a control cotton boll that exhibits fluorescent marks very similar to stink bug puncture marks. Those marks would typically be identified as positives and count as false positives. (**G**) and (**H**) are images of a cotton boll with exterior puncture marks caused by stink bug feeding. The marks are visible as larger fluorescent green regions and count as true positives. Note the presence of smaller fluorescent dots not caused by stink bugs. The scale bar (white line in lower left corner of image A) represents 5 mm.

The image processing method was developed based on the observation that (a) puncture marks cause a circular limited region where green fluorescence develops, and (b) the center of the region, where the plant tissue was removed by the puncture, actually exhibited very low fluorescence, leading to a donut-shaped intensity pattern. Examples of the image patterns are shown in Figure 5, both under normal fluorescence and after processing to yield the ratiometric image.

One challenge for the image analysis method is the presence of fluorescent regions that are not related to stink bug feeding (Figure 5E and F). In fact, out of 109 control cotton bolls that were not exposed to stink bugs, 41 showed fluorescent marks, and a total of 49 fluorescent marks met the inclusion criteria and were included in the analysis. Conversely, out of 136 cotton bolls that were exposed to stink bugs, 38 did not show any fluorescent marks, and manual examination of the inner carpal wall and lint confirmed that these bolls had not been fed upon by the stink bugs (i.e., true negatives). Of the remaining 98 cotton bolls that exhibited fluorescent marks, a total of 169 marks were included in the analysis. Manual examination of cotton bolls not included in this study led to the inclusion criteria, namely, a minimum area, a minimum average intensity, and a strictly convex shape. It is conceivable that small marks are caused by non-piercing insects, such as spider mites [22], and large, elongated marks are the consequence of mechanical abrasion.

Clearly, any image analysis method must detect false-positive fluorescent marks. The first step of the image analysis chain was aimed at detecting all fluorescent marks, irrespective of their origin. Local maximum detection combined with either gradient-based segmentation (e.g., the hill-climbing algorithm [23] or active contours (“snakes” [20]) or intensity-based segmentation, such as region-growing, can be used in a straightforward manner to provide the initial set of candidates. Based on the initial set of cotton bolls, we defined all fluorescent marks that were found on the control group of cotton bolls as false-positives, and all fluorescent marks that were found on the exposed group of cotton bolls as potential true positives. The fluorescent marks associated with needle punctures (total of 81 marks) were examined as a separate group.

**Figure 6:**
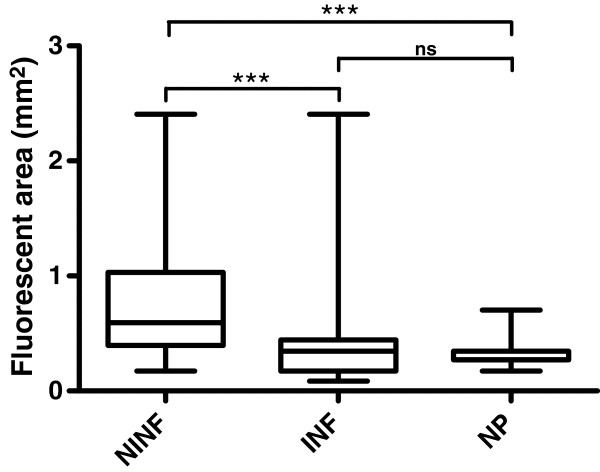
**Boxplot of the area *A* of the segmented fluorescent region for three categories of fluorescent spots: non-infested (NINF), infested (INF), and needle punctures (NP).** Median area is significantly larger (*P *< 0*.*0001) for fluorescent areas that are neither stink bug-related nor caused by needle puncture, but the area is not significantly different between stink bug feeding marks and needle punctures (Kruskal-Wallis test with Dunn’s multiple-comparison post-test).

The second step of the image analysis process, which was the focus of this study, was aimed at separating true positives from false positives. Among several quantitative metrics, size had a strong ability to separate false and true positives as shown in Figure 6. The median area of the fluorescent region associated with stink bug feeding was 0.35mm^2^ compared to a median area of 0.6mm^2^ for the false positives. The difference was statistically significant (*P *< 0*.*0001). Needle puncture marks caused an even smaller fluorescent region with a median area of 0.27mm^2^. Size variation was less within the marks caused by stink bug feeding and those caused by the needle puncture, whereas a large variability in size was observed in the false-positive group. Median size was not statistically different between stink bug-related marks and needle puncture marks.

A second quantitative metric was obtained by measuring the intensity drop-off towards the center of the puncture mark. False-positive marks tend to have an irregular intensity distribution with the highest intensity near the center. Conversely, plant tissue that was directly damaged by the puncture was almost black under visible-light examination, and its corresponding fluorescent emission lower. It can therefore be expected that an intensity ratio of the center intensity *I*_1_, relative to the average intensity of the entire fluorescent spot *I*_2_, tends to be less than 1 for true-positives and greater than 1 for false-positives. In some cases (such as the one displayed in Figure 5G and H), the dark center region was not as pronounced as in most cases, and we accepted an intensity ratio of *I*_1_/*I*_2 _< 1*.*2 as indication of a positive (i.e., stink bug-caused) fluorescent mark.

**Figure 7:**
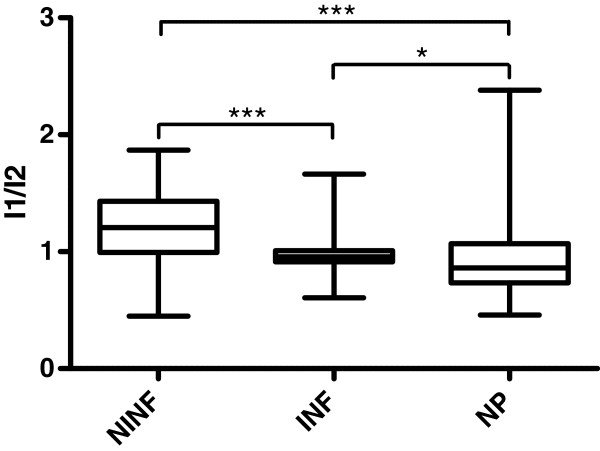
**Boxplot of the intensity ratio *I***_**1**_**/*I***_**2 **_**for three categories of fluorescent spots: non-infested (NINF), infested (INF), and needle punctures (NP).** Fluorescent marks that are not related to a puncture generally have a higher intensity near the center. Correspondingly, the ratio *I*_1_/*I*_2 _is greater than 1 (statistically significant, Wilcoxon signed rank test, *P *< 0*.*0001). Puncture marks tend to have a darker center, and the ratio *I*_1_/*I*_2 _is less than 1. This difference is also statistically significant for stink bug feeding marks and needle punctures. Furthermore, the ratio *I*_1_/*I*_2 _is significantly higher for fluorescent marks that are not related to a puncture than for those related to stink bug feeding and those related to needle punctures (both *P *< 0*.*0001 by the Kruskal-Wallis test with Dunn’s multiple-comparison post-test).

A box plot of the intensity ratio can be seen in Figure 7. Median ratiometric intensity of the false-positive fluorescent regions was 1.2, with a statistically significant difference from 1 (Wilcoxon signed rank test, *P *< 0*.*0001). Conversely, median ratiometric intensity of those fluorescent regions associated with stink bug damage was 0.96, statistically significant lower than 1 (Wilcoxon signed rank test, *P *< 0*.*0001). For comparison, needle punctures exhibited a median ratiometric intensity of 0.86, lower than 1 with *P *= 0*.*0002. Similar to size, the ratiometric intensity was significantly different between false positives and true positives (*P *< 0*.*0001) and between false positives and needle puncture marks (*P *< 0*.*0001).

It is interesting to note the relative similarity between fluorescent marks caused by stink bug feeding and those caused by needle puncture, which is consistent with our earlier publication [18], where we hypothesized that the fluorescence emission is the by-product of the plant healing process. Equally relevant is the observation that the fluorescent marks do not fade over a period of seven days (i.e., the maximum period over which we examined a cotton boll). This finding confirms those we reported earlier [18].

**Figure 8:**
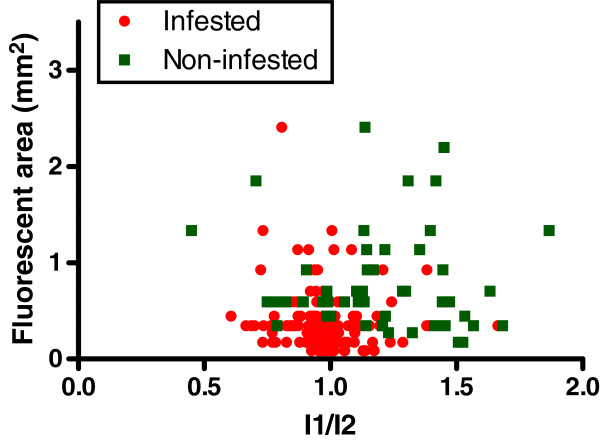
**Scatterplot of all examined marks (except the needle puncture controls) arranged in two dimensions by area *A* and intensity ratio *I***_**1**_**/*I***_**2**_**.** A multiple-threshold criterion, for example *I*_1_/*I*_2 _< 1*.*2 and *A *< 0*.*6, improves the separation of false-positive marks from true-positive marks: 87% of the true-positive marks lie inside the rectangle, whereas only 8% of the false-positives fall into the same rectangular region.

Although both the size metric and the intensity ratio metric are different between stink bug feeding marks and unrelated fluorescent spots from a statistical perspective, considerable overlap between the values of the true positive and false positive groups exists. We therefore examined if a two-dimensional separation can further improve the separation of false and true positives. A scatter plot of both groups in two dimensions is shown in Figure 8. Overlap still exists, but it can be envisioned that a rectangle that separates the lower left corner from the rest of the region can capture most of the true positives. Such a rectangle could, for example, combine the conditions *I*_1_/*I*_2 _< 1*.*2 and *A *< 0*.*6. Puncture marks from infested cotton bolls have a ratio *I*_1_/*I*_2 _< 1 in 69% of the cases, whereas for false-positive damage the ratio falls below one in only 24% of the cases. Only four (8%) false-positives lie within the rectangle arbitrarily defined above, and 147 true-positives (87%) lie within the same rectangle. Contingency tables for the criteria cited above are provided in Table 1. Clearly, two-dimensional separation markedly improves the specificity of the detection method.

**Table 1:** **Contingency tables for separation of fluorescent marks by their area *A*, their intensity ratio *I***_**1**_**/*I***_**2**_**, and both criteria simultaneously**

	***A *< 0*.*6**	***A *≥ 0*.*6**	$\frac{I_{1}}{I_{2}}<1.2$	$\frac{I_{1}}{I_{2}}\geq 1.2$	***A *< 0*.*6 and**	***A *≥ 0*.*6 or**
					$\frac{I_{1}}{I_{2}}<1.2$	$\frac{I_{1}}{I_{2}}\geq 1.2$
Infested *n *= 169	70.2%	7.3%	73.9%	3.7%	67.4%	10.6%
Control *n *= 49	12.4%	10.1%	11.0%	11.5%	6.0%	16.5%

**Figure 9:**
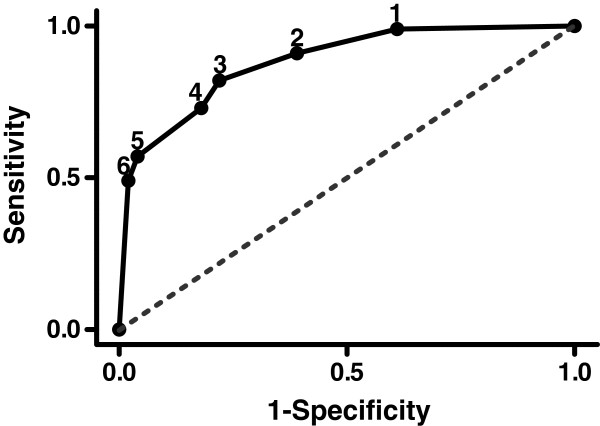
**Receiver operating characteristic (ROC) for different-size rectangles (*cf.* Figure**8**) as a study how well a dual threshold criterion can separate true and false positive fluorescent marks.** As specific corner points for the rectangle in Figure 8 were chosen: 1: (1.5, 1.4), 2: (1.0, 1.2), 3: (0.8, 1.1), 4: (0.65, 1.05), 5: (0.5, 1.0), 6: (0.4, 0.98). At point 1, sensitivity is high but specificity is low, and this changes towards the higher point number. At point 6 we reach lower values for sensitivity but higher values for specificity. Striking a balance in this instance includes reducing the number of false positives (high sensitivity) and false negatives (high specificity). Dashed line represents a random assumption.

In an attempt to determine the limits of separation between true and false positives, a receiver operating characteristic (ROC) analysis was performed by increasing the size of the rectangle along a diagonal through the origin and the point (0.6, 1.2). As shown in Figure 9, choice of a very small rectangle leads to high sensitivity, but poor specificity due to the exclusion of many true positives. Too large a rectangle leads to poor sensitivity due to the inclusion of the false positives. The ROC curve, however is very different from the diagonal (i.e., assumption of random data). The ROC analysis does not take into account that different separation boundaries (e.g., a diagonal line connecting *A *= 1*.*1 and *I*_1_/*I*_2 _= 1*.*5, or an ellipse) can improve both sensitivity and specificity even further. These considerations are not appropriate for this stage of the research, however, because a larger-scale study is necessary to identify data obtained in the field. It is conceivable that clustering algorithms can provide the boundary from a training data set, and points placed in the two-dimensional coordinate system can be assigned to their respective cluster.

For practical purposes, an 87% detection of true positives with only 8% false-positives is already acceptable when we consider that the main goal of the application of this method in the field is the approximate determination of the level of stink bug infestation. The reason for this conclusion lies in the study by Reay-Jones [24] who found an average damage rate of 14.8% on a scale from 0% to 100% in a very large sample from commercial cotton fields in Georgia and South Carolina. Moreover, Extension-recommended treatment thresholds range from 15% to 30% internal boll damage, depending on week of bloom [25]. In our previous study of the new fluorescent method [18], accuracy for the conventional visual inspection was found to be 75%, while the accuracy for the florescent detection method was 92.9% with a 7.7% false positive rate when a human observer analyzed the fluorescent images.

A concurrent development of a method to detect stink bug presence and damage to cotton bolls is an ”electronic nose” sensor for specific volatiles emitted by stink bugs or damaged plants [26]. Under laboratory conditions, the internal boll injury (interior walls of bolls with warts and lint damage) was predicted in 95% of all cases [26], and the electronic nose was able to identify injured and healthy cotton bolls with an accuracy between 80 and 90% [27]. Notably, the detection accuracy of 84% reported in this study is in a similar range as the one reported by Degenhardt *et al.*[27].

In our previous work [18], we identified a fluorescent wand and a fluorescent image scanner as possible devices to detect stink bug-related fluorescence. Further analysis of the fluorescent marks, as presented in this study, reveals that the wand is likely impractical due to the presence of a large number of false-positive fluorescent marks. The second device that we proposed is a light-shielded enclosure in which fluorescence emission can be detected by pushing the cotton boll into the enclosure. With a suitably engineered device, the cotton boll might even be left on the plant. Irrespective of the final embodiment of a field-deployable device, we can expect a significant increase of cotton boll throughput and analysis speed. Based on the study by Reay-Jones [24], the appropriate sample size for arriving at a treatment decision is 15 samples (20 bolls per sample) under the assumption of a boll injury rate of 14.3% and the accepted error rate of 30% of the mean. Toews et al. [16] compared the time required for internal examination of bolls and found that a single 20-boll sample required 7 minutes to collect and process. Since field scouts are unable to invest the required time to reach this level of precision, they tend to collect fewer bolls and consequently accept a much higher error rate. The fluorescent detection promises much faster sampling as image acquisition and analyses could be performed instantaneously. Examining more bolls in less time promises a more accurate estimate of boll damage. These data strongly support that the fluorescent detection method is a major step forward in making more accurate estimates of stink bug damaged bolls for making pest management decisions.

## Conclusions

Among the many challenges facing agronomists in growing cotton, two stand prominently: detection of internal damage and determination of its underlying cause. With respect to cotton, current methods include harvesting immature cotton bolls and opening them so any damage can be visualized. Even if no damage is observed, the cotton bolls are lost irretrievably due to destructive sampling. Furthermore, small bolls are nearly always shed by the plant as a result of stink bug feeding [28]. In that case, the damaged bolls would not be included in the samples, because they would have been dropped by the plant before reaching appropriate diameter for sampling. It is also desirable to detect boll damage very quickly so that the stink bug population can be mitigated with insecticides before the bugs move to new hosts.

To overcome these limitations, we present one step towards a field applicable imaging method to estimate the levels of stink bug infestation. The next step of this project is the engineering design of an imaging scanner that can be used in the field. We envision a hand-held imaging apparatus, where the illumination source and an image sensor array are placed in an enclosure. A cotton boll that is placed in this enclosure needs to be shielded from environmental light. One desirable property of this enclosure that allows the boll to stay on the plant, although sampling by harvesting some bolls and inserting them in the imaging apparatus may turn out to be a suitable alternative. An additional motivation for developing a device that can analyze the boll on the plant is its use in research to examine fluorescent marks and stink bug damage over a longer period of time.

Either way, with the image analysis methods described herein, the theoretical foundations towards a field-usable detection device for stink bug damage in cotton bolls are laid.

## Competing interests

The authors declare that they have no competing interests.

## Authors’ contributions

MAH and MT made the original discovery of stinkbug-related fluorescence. MT provided the controlled set of cotton bolls. MAH designed the imaging apparatus and parts of the image analysis chain. AM and MAH developed the intensity ratio metric. AM and EER acquired and processed all photographic and microscopic images, and performed the statistical analysis. All authors contributed to the data analysis and to the manuscript. All authors read and approved the final manuscript.
